# Baseline cognitive performance moderates ACL injury–related knee biomechanics during unanticipated sidestep cutting in highly trained male soccer players

**DOI:** 10.3389/fphys.2026.1911093

**Published:** 2026-08-18

**Authors:** Weipeng Liu, Zilong Wang, Zhuangzhuang Wang, Zhiyuan Qin, Zhengwei Wu, Weihua Zheng, Congjie Cheng, Weichao Li, Wenhua Li

**Affiliations:** 1 School of Strength and Conditioning, Shenyang Sport University, Shenyang, China; 2 School of Physical Education, Jimei University, Xiamen, China; 3 School of Social Sports, Shenyang Sport University, Shenyang, China; 4 College of Exercise and Health, Shenyang Sport University, Shenyang, China

**Keywords:** anterior cruciate ligament injury, change of direction, cognitive–motor interaction, football, neurocognitive performance

## Abstract

**Objective:**

To compare anterior cruciate ligament (ACL) injury–related knee biomechanics between anticipated and unanticipated sidestep cutting and to investigate whether baseline cognitive performance moderated these biomechanical responses in highly trained male soccer players.

**Methods:**

Thirty highly trained male soccer players completed a computerized Go/No-Go task to assess inhibitory control, including reaction time (RT), commission error rate (CER; responses incorrectly made during No-Go trials), and omission error rate (OER; failures to respond during Go trials). Participants subsequently performed anticipated and unanticipated 90° sidestep cutting tasks while three-dimensional motion capture and force plate data were simultaneously collected. Peak knee flexion angle, peak knee abduction angle, peak knee abduction moment, peak anterior tibial shear force (ATSF), peak vertical ground reaction force (vGRF), and loading rate were analyzed. Paired comparisons were first performed to examine overall biomechanical differences between the anticipated and unanticipated conditions. Linear mixed-effects models were then used to investigate whether cognitive performance moderated the biomechanical responses to task anticipation.

**Results:**

Compared with the anticipated condition, unanticipated sidestep cutting resulted in greater peak knee flexion angle (*d* = 0.389, 95% CI, 0.118–0.660, *p* = 0.006), peak knee abduction angle (*d* = 0.339, 95% CI, 0.189–0.489, *p* < 0.001), and peak knee abduction moment (*d* = 1.481, 95% CI, 0.857–2.104, *p* < 0.001). Significant anticipation × RT interactions were observed for peak knee abduction angle (*β*_std_ = 0.156, 95% CI, 0.039–0.274, *p* = 0.031), peak knee abduction moment (*β*_std_ = 0.380, 95% CI, 0.168**–**0.592, *p* < 0.001), and peak ATSF (*β*_std_ = 0.517, 95% CI, 0.350–0.684, *p* < 0.001). Significant anticipation × CER and anticipation × OER interactions were observed for peak knee abduction moment (*β*_std_ = 0.504, 95% CI, 0.299–0.710, *p* < 0.001) and peak vGRF (*β*_std_ = 0.463, 95% CI, 0.245–0.682, *p* < 0.001), respectively.

**Conclusion:**

Unanticipated sidestep cutting altered multiple ACL injury–related biomechanical variables in highly trained male soccer players. More importantly, athletes’ baseline cognitive performance moderated several ACL injury–related biomechanical responses.

## 1 Introduction

During soccer match play, players frequently decelerate, plant the foot, and redirect their center of mass to achieve technical or tactical objectives. Sidestep cutting is a common change-of-direction maneuver used during attacking, defending, pressing, and opponent evasion (Kono et al., 2025). Soccer players perform approximately 305 ± 50 change-of-direction actions per match, nearly 77% of which occur at angles ≤90° (Morgan et al., 2022). Although fundamental to performance, sidestep cutting imposes substantial mechanical demands on the knee, particularly at sharper angles (Li and Qian, 2025; Dos’Santos et al., 2021a; Yu and Garrett, 2007). Compared with 45°cutting, 90°cutting increases peak knee abduction moment by 0.36 N·m·kg^-^¹ and peak knee abduction angle by 2.7°, both of which are associated with anterior cruciate ligament (ACL) injury mechanisms (Dos’Santos et al., 2021a; Kristianslund et al., 2014). Consistent with these biomechanical demands, approximately 26–67% of ACL injuries in soccer occur during cutting or change-of-direction actions (Sundberg et al., 2025). More importantly, among soccer-related injuries, ACL injuries impose one of the greatest burdens on players, with return to play typically requiring approximately 9 months (Hallén et al., 2024; Liu et al., 2026a).

In open-skill sports such as soccer, athletes frequently execute movements in unpredictable environments under strict time constraints, requiring rapid perception and integration of dynamic visual, auditory, and proprioceptive information (Jiménez-Martínez et al., 2025a). Recent systematic reviews have consistently shown that, compared with pre-planned cutting, unanticipated and dual-task sidestep cutting result in greater ACL injury–related biomechanical responses, including increased knee abduction angle, knee abduction moment, and vertical ground reaction force (vGRF), with these risks further exacerbated under higher cognitive task demands (Ebner et al., 2025; Xu et al., 2025; Jiménez-Martínez et al., 2025a). Available reaction time (ART) represents a key modulating factor influencing movement preparation under unanticipated conditions. A previous study demonstrated that reducing ART from 850 ms to 500 ms disrupted anticipatory postural adjustments and constrained feedforward movement planning prior to foot contact (Mornieux et al., 2014). Collectively, these findings suggest that ACL injury-related biomechanics during sidestep cutting cannot be explained solely by mechanical or neuromuscular factors.

Accordingly, increasing attention has been directed toward the role of cognitive performance in musculoskeletal injury susceptibility (Avedesian et al., 2022; Bertozzi et al., 2023b; Herman et al., 2015). Cognitive function refers to a set of mental processes involved in the perception, processing, and response to environmental information, including visual attention, self-monitoring, processing speed, reaction time (RT), working memory, and response inhibition, all of which are essential for dynamic motor planning and decision-making in sporting contexts (Herman et al., 2015; Diamond, 2013). Recent work demonstrated that increased cognitive demands during a single-leg drop jump task altered knee biomechanics, with athletes exhibiting poorer cognitive performance showing less favorable movement patterns associated with ACL injury mechanisms (Jiménez-Martínez et al., 2026). This cognitive–motor interaction provides a plausible explanation for why injuries frequently occur during cognitively demanding tasks.

Although a growing body of evidence suggests that poorer cognitive performance is associated with less favorable ACL injury-related biomechanics (Bertozzi et al., 2023b; Jiménez-Martínez et al., 2026; Bertozzi et al., 2023a), evidence from highly trained athletes (i.e., Tier 3–4 athletes) remains limited (McKay et al., 2022). On the one hand, highly skilled athletes may make more effective decisions and process environmental information more efficiently than their less-skilled counterparts, which may facilitate the adoption of safer movement strategies (Kipp et al., 2013). On the other hand, highly trained athletes typically perform sidestep cutting at faster approach and exit velocities and with shorter ground contact times (McBurnie et al., 2021). Although these characteristics are advantageous for performance, they may also increase mechanical loading and contribute to the well-documented “performance–injury conflict” (Dos’Santos et al., 2021b). To address this gap, the present study examined whether baseline cognitive performance moderated knee biomechanical responses to anticipated and unanticipated sidestep cutting in highly trained male soccer players.

Therefore, the present study had two aims: first, to compare ACL injury-related knee biomechanical variables between anticipated and unanticipated sidestep cutting in highly trained male soccer players; and second, to determine whether individual differences in baseline cognitive performance remain relevant to movement control under unanticipated conditions even among highly trained athletes. We hypothesized that unanticipated sidestep cutting would elicit less favorable ACL injury-related biomechanical patterns than anticipated sidestep cutting. Furthermore, we hypothesized that athletes with poorer baseline cognitive performance would exhibit more pronounced unfavorable biomechanical responses under unanticipated conditions.

## 2 Materials and methods

### 2.1 Participants

This study was conducted in accordance with the Declaration of Helsinki and approved by the Ethics Committee of Shenyang Sport University (Approval No. 202631). All participants were informed of the experimental procedures, and potential risks and provided written informed consent prior to participation. An *a priori* power analysis was conducted using GLIMMPSE software (version 3.1.3, University of Florida and University of Colorado Denver, USA) to determine the required sample size (Kreidler et al., 2013). The analysis was conducted using a repeated-measures design with the Hotelling–Lawley trace test, with statistical power set at 80% and the Type I error rate set at 0.05. The results indicated a minimum required sample size of 18 participants. Peak knee flexion angle, peak knee abduction angle, and peak knee abduction moment were selected as outcome variables, with mean values derived from previous studies comparing anticipated and unanticipated sidestep cutting (Kim et al., 2014; Lei and Cheng, 2025).

Participants were eligible if they met the following criteria: (1) highly trained male soccer players competing at the national level (Tier 3) (McKay et al., 2022); (2) no history of ACL injury or lower-extremity surgery, no lower-extremity musculoskeletal injury requiring medical treatment, and no concussion within the past six months; (3) no diagnosed neurological, cardiovascular, visual, or psychiatric disorders that could potentially impair cognitive performance or motor control; (4) able to perform anticipated and unanticipated sidestep cutting tasks without pain or movement restrictions; and (5) right-leg dominant, defined as the preferred leg used to kick a ball. Conversely, participants were excluded if they reported pain during testing, failed to complete the cognitive assessment or cutting protocol, or were unable to produce valid biomechanical data due to technical or procedural issues.

Considering typical attrition rates in sports science research, a total of 30 male soccer players were recruited for the study. Participants’ demographic characteristics are summarized in **Table 1**. All participants were recruited from the National Youth School Football League in China (Men’s University Division A), and data collection was conducted during the off-season.

**Table 1:** Demographic characteristics and cognitive performance of participants.

Value (*n* = 30)	
Age (y)	21.10 ± 2.04
Height (cm)	178.73 ± 5.51
Weight (kg)	72.3 ± 7.87
Training experience (y)	9.03 ± 2.34
RT (ms)	292.05 ± 22.30
CER (%)	5.00 [1.67–11.67] (0.00–16.67)
OER (%)	1.67 [0.56–3.33] (0.00–6.11)

Values are presented as mean ± SD or median [IQR] (range).

RT, reaction time; CER, commission error rate; OER, omission error rate.

### 2.2 Experimental design

This study employed a controlled laboratory design. Participants visited the laboratory on three separate occasions. During the first visit, participants were familiarized with the experimental equipment and practiced the required tasks until they could perform them proficiently and demonstrated a clear understanding of the procedures. During the second visit, participants completed a baseline cognitive assessment using the Go/No-Go task. The third visit was conducted more than 48 h after the second visit, during which participants performed anticipated and unanticipated sidestep cutting tasks while biomechanical data were simultaneously collected. The order of the two cutting conditions was randomized using a computer-generated sequence to minimize potential order effects. For each participant, all laboratory visits were scheduled at the same time of day to minimize potential circadian effects. All testing was conducted in a temperature- and humidity-controlled laboratory maintained at 22–24 °C and 40%–60% relative humidity. Before each visit, participants were instructed to refrain from strenuous exercise and from consuming alcohol and caffeine consumption for 24 h and to obtain at least 7 h of sleep before testing.

#### 2.2.1 Go/No-go task

Response inhibition was assessed using a computerized Go/No-Go task administered with E-Prime software (Version 3.0, Psychology Software Tools, USA). Impaired response inhibition has been suggested to be potentially associated with an increased risk of non-contact ACL injury in soccer players (Gokeler et al., 2024). The task design was adapted from previous studies and employed grey geometric shapes as visual stimuli (Ludyga et al., 2021, 2023). The outlines of the shapes were presented in yellow, pink, or blue, corresponding to frequent Go trials (40%), standard Go trials (30%), and No-Go trials (30%), respectively. Frequent Go trials were included to establish a prepotent response tendency, thereby increasing the response-inhibition demands of the No-Go trials. Participants were instructed to press the space bar as quickly and accurately as possible in response to frequent Go and standard Go trials and to withhold a response to No-Go trials. Each stimulus was presented for 150 ms, followed by an 850 ms response window. To minimize anticipatory responding, the interstimulus interval varied randomly between 1200 and 1400 ms. Before the formal test, participants completed 30 practice trials to ensure adequate understanding of the task requirements. The formal assessment consisted of three blocks of 100 trials, resulting in a total of 300 trials. For statistical analyses, mean RT was calculated from correct standard Go trials because their presentation probability was equivalent to that of No-Go trials, thereby reducing potential stimulus-frequency-related facilitation. Responses shorter than 150 ms were considered anticipatory responses and excluded from the RT calculation (Galloway-Long et al., 2022). Commission error rate (CER) was defined as the proportion of No-Go trials in which participants pressed the space bar, whereas omission error rate (OER) was defined as the proportion of Go trials in which participants failed to respond within the response window. After invalid trials were excluded, all valid trials across the three blocks were pooled to calculate each participant’s mean RT, CER, and OER.

#### 2.2.2 Sidestep cutting task

**Figure 1:**
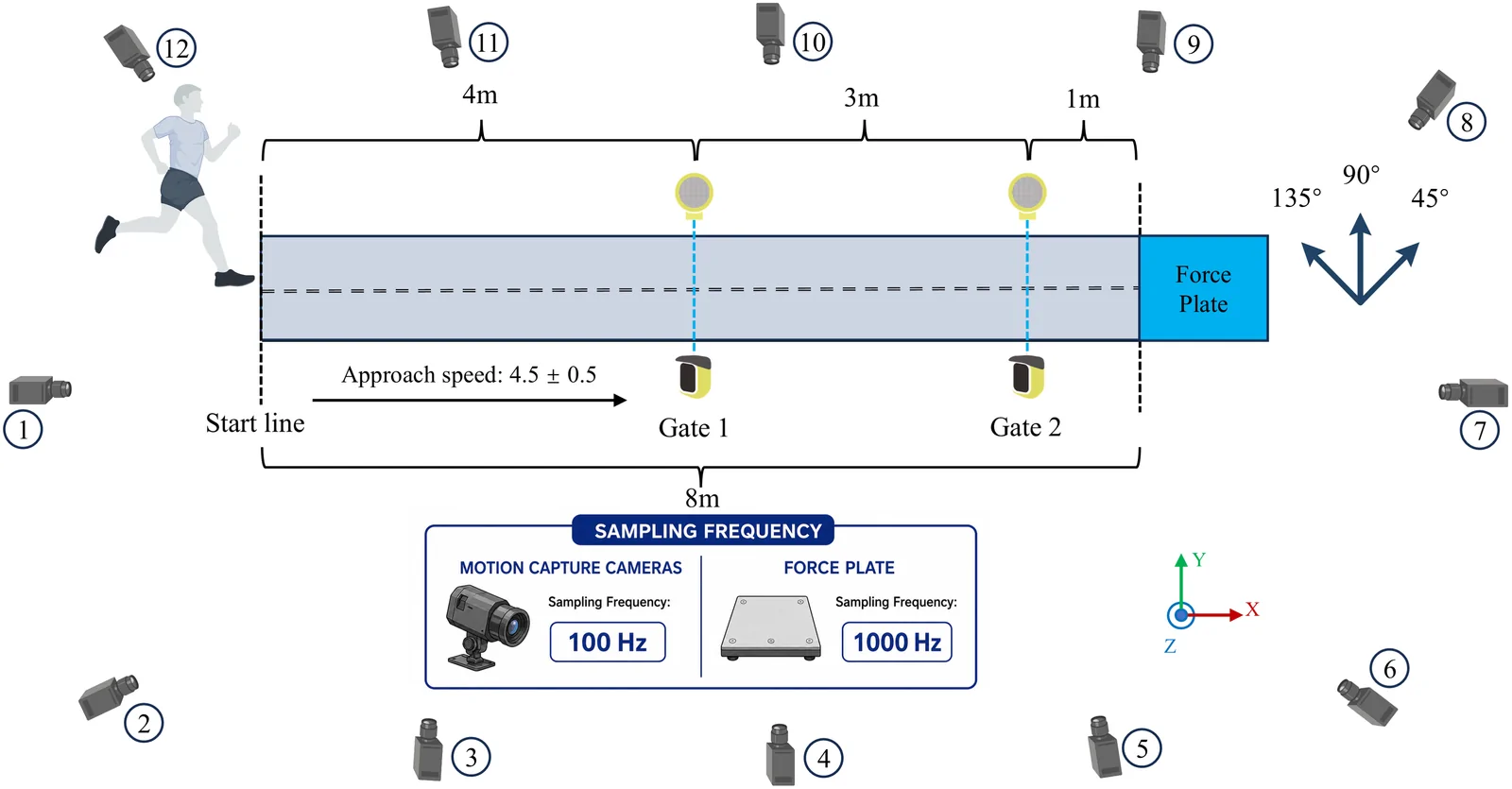
Schematic of the experimental layout for the sidestep cutting task.

Prior to data collection, participants changed into standardized laboratory attire (Nike Pro compression shorts, Nike, Beaverton, OR, USA) and athletic shoes (Nike Air Zoom Pegasus 39, Nike, Beaverton, OR, USA), and completed a 10 min standardized warm-up. Participants performed sidestep cutting trials following an 8 m approach run on a wooden floor covered with anti-slip mats (**Figure 1**). A single-beam timing system (Witty, Microgate, Italy), positioned approximately at hip height, was used to control the approach speed at 4.5 ± 0.5 m/s (Lee et al., 2013; Liu et al., 2026b).

During unanticipated sidestep cutting, participants were required to respond to visual cues presented on a monitor positioned directly in front of them. Specifically, the first timing gate was positioned 4 m before the force plate. Four hundred milliseconds after participants passed through this gate, an arrow indicating the required cutting direction was randomly presented on the monitor (45°, 90°, or 135° to the left). The presentation frequency and sequence of the three directional cues were randomly determined using the built-in randomization function of the Witty system, with no restrictions on consecutive presentations of the same direction. Based on the approach velocity, ART ranged from 400 to 600 ms, thereby imposing sufficiently strict time constraints on the participants (Ebner et al., 2025). During anticipated sidestep cutting, participants were required to perform only a pre-planned 90° sidestep cut to the left.

A trial was considered successful when participants approached within the required speed range, completed the sidestep cutting maneuver in the instructed direction, maintained balance throughout the movement, and landed entirely on the force plate without contacting its boundaries. Trials that did not meet any of the predefined success criteria were excluded and repeated. Participants were given 30 s of rest between trials and a 10 min rest interval between the anticipated and unanticipated conditions to minimize the potential influence of fatigue on biomechanical performance. For each condition, testing continued until the first three successful 90° trials were obtained, and these trials were included in the biomechanical analysis. Participants completed an average of 3.76 ± 0.72 trials in the anticipated condition and 6.86 ± 1.56 trials in the unanticipated condition to obtain the required three successful 90° trials. The 90°cutting angle was selected because it frequently occurs during soccer match play and has been associated with greater ACL injury-related biomechanical loading than other cutting angles (Bloomfield et al., 2007; Dos’Santos et al., 2021a; Morgan et al., 2022). The 45° and 135° directions were included as alternative response options to maintain directional uncertainty and increase the choice-response demands of the unanticipated task, but were not included in the biomechanical analysis.

### 2.3 Data acquisition

Kinematic data were collected using a 12-camera infrared motion capture system (Vantage V5, Vicon Motion Systems, UK), and GRF data were simultaneously recorded using an in-ground force plate (9287CA, Kistler Instrumente, Switzerland) at sampling frequencies of 100 Hz and 1000 Hz, respectively. Twenty-eight retro-reflective markers (14 mm in diameter) were attached to anatomical landmarks according to the Conventional Gait Model 2.3 (Wang et al., 2024). All markers were placed by the same experienced researcher to ensure consistency.

### 2.4 Data processing

The raw data were imported into Visual3D software (Version 6, C-Motion, USA) for kinematic and inverse dynamics analyses. Joint angles were calculated using an X–Y–Z Cardan rotation sequence, with the X-, Y-, and Z-axes corresponding to flexion–extension, abduction–adduction, and internal–external rotation, respectively. To minimize the influence of impact artifacts and avoid artificially inflated joint moments during inverse dynamics analyses, marker trajectories and force data were filtered using a zero-lag, fourth-order Butterworth low-pass filter with an identical cutoff frequency of 15 Hz (Kristianslund et al., 2014, 2012). The filtered ground reaction force data were subsequently used for inverse dynamics calculations and the derivation of kinetic variables.

A region-of-interest approach was used to define the weight acceptance (WA) phase during sidestep cutting, which has been considered the phase associated with the greatest risk of ACL injury (Zago et al., 2024; Lee et al., 2013; Pataky et al., 2016). Initial contact (IC) was defined as the instant at which vGRF exceeded 10 N. The WA phase was defined as the period from IC to the first trough of the unfiltered vGRF signal. If no distinct trough was observed, the first 30% of the stance phase was defined as the WA phase (Donnelly et al., 2017).

The ACL injury–related biomechanical variables of interest during the WA phase included: (1) peak knee flexion angle, defined as the maximum sagittal-plane rotation of the right shank relative to the right thigh, with flexion defined as positive (°); (2) peak knee abduction angle, defined as the maximum frontal-plane rotation of the right shank relative to the right thigh, with knee abduction defined as positive (°); (3) peak knee abduction moment, defined as the maximum frontal-plane knee joint moment calculated using inverse dynamics, expressed in the local coordinate system of the right thigh, with the external knee abduction direction defined as positive, and normalized to body mass (N·m·kg^-^¹); (4) peak anterior tibial shear force (ATSF), defined as the maximum anteriorly directed knee joint reaction force calculated using inverse dynamics, expressed in the local coordinate system of the right shank, with the anterior direction defined as positive, and normalized to body mass (N·kg^-^¹); (5) vGRF, defined as the maximum vertical ground reaction force recorded by the force plate, with the upward direction defined as positive, and normalized to body weight (BW); and (6) vertical loading rate (vLR), calculated using the formula (BW·s^-^¹):


$$\text{vLR}=\frac{\text{Peak vGRF}}{\text{Time to peak vGRF}}$$

### 2.5 Statistical analysis

All statistical analyses were performed in R (Version 4.4.3, R Foundation for Statistical Computing, Austria) using the effsize, lme4, lmerTest, MuMIn, and emmeans packages, with statistical significance set at *α* = 0.05. Descriptive statistics were presented as mean ± standard deviation (SD). For paired comparisons, biomechanical variables were first averaged across successful trials within each condition for each participant to compare biomechanical outcomes between anticipated and unanticipated sidestep cutting. The normality of the paired differences was assessed using the Shapiro–Wilk test, followed by paired-samples *t*-tests. Cohen’s *d* effect sizes with 95% confidence intervals (CIs) were calculated and interpreted as: trivial (< 0.20), small (0.20–0.59), moderate (0.60–1.19), large (1.20–1.99), and very large (≥ 2.0) (Hopkins et al., 2009).

Subsequently, exploratory linear mixed-effects models (LMMs) were constructed to examine whether individual differences in cognitive performance moderated biomechanical responses during sidestep cutting. LMMs account for correlated observations in repeated-measures designs by modeling intra-individual variability through participant-level random effects (Meteyard and Davies, 2020). To minimize potential multicollinearity among cognitive metrics, separate LMMs were constructed for each of the six biomechanical outcomes and for each cognitive metric (RT, CER, and OER), resulting in a total of 18 models. In each model, anticipation (anticipated vs. unanticipated), the cognitive metric, and their interaction were included as fixed effects, with participant included as a random intercept to account for within-subject variability. Models were fitted using restricted maximum likelihood estimation, and degrees of freedom and *p*-values for the fixed effects were estimated using the Satterthwaite approximation as implemented in the lmerTest package. Standardized regression coefficients (*β*_std_), 95% CIs, and *p*-values were reported for all fixed effects. Model fit was evaluated using marginal *R*² (*R*²_m_) and conditional *R*² (*R*²_c_) (Ives, 2019). Numerical convergence and boundary-fit issues were assessed by examining optimizer convergence codes, maximum relative gradients, and singular-fit diagnostics. Residual normality was assessed separately for each model using residual skewness and excess kurtosis.

RT, CER, and OER were measured once per participant and were therefore treated as participant-level predictors based on 30 individuals, whereas the 180 biomechanical trials represented repeated observations nested within participants. Prior to analysis, all continuous variables were standardized using z-scores to facilitate interpretation and comparison of standardized regression coefficients across cognitive metrics, biomechanical outcomes, and models. Peak knee abduction moment was predefined as the primary biomechanical outcome, and the corresponding *p*-values were reported without adjustment. The remaining five biomechanical outcomes were considered secondary. For each cognitive metric (RT, CER, and OER), the *p*-values for the anticipation effect, cognitive-metric effect, and anticipation × cognitive-metric interaction across these five secondary outcomes (15 tests in total) were adjusted simultaneously using the Benjamini–Hochberg false discovery rate procedure. Simple-slope analyses were performed when significant anticipation × cognitive-metric interactions were observed, with standardized regression coefficients, 95% CIs, and *p*-values reported separately for the anticipated and unanticipated conditions.

## 3 Results

### 3.1 Anticipation effects on knee biomechanics

**Figure 2:**
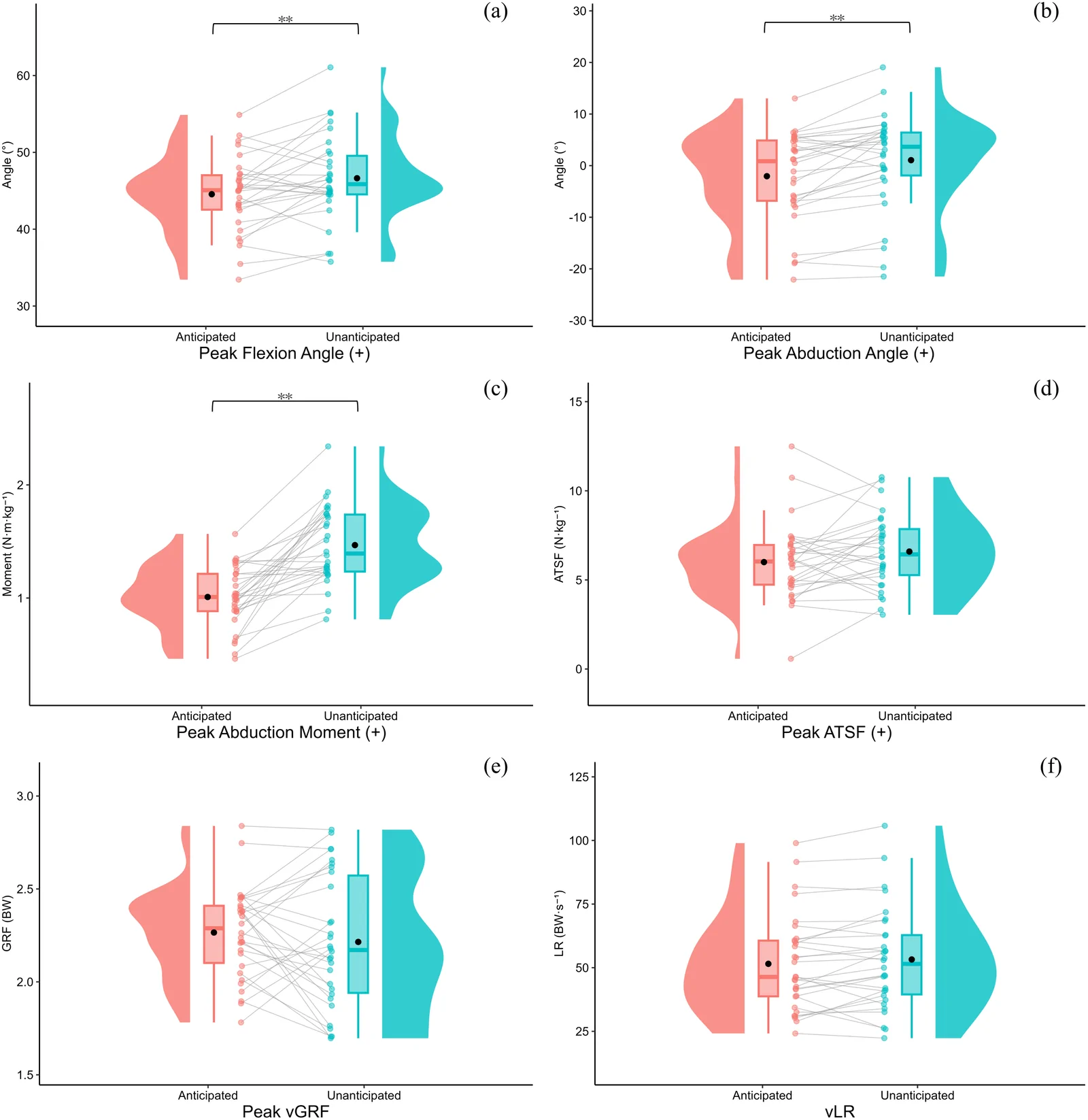
Raincloud plot of anticipation on knee biomechanics during sidestep cutting. **(a)** Peak flexion angle; **(b)** Peak abduction angle; **(c)** Peak abduction moment; **(d)** Peak ATSF; **(e)** Peak vGRF; **(f)** vLR. **p < 0.01: significant differences between anticipated and unanticipated sidestep cutting. ATSF, anterior tibial shear force; vGRF, vertical ground reaction force; vLR, vertical loading rate.

Significant differences in knee biomechanics were observed between anticipated and unanticipated sidestep cutting conditions (**Table 2**; **Figure 2**). For knee kinematics, participants demonstrated greater peak knee flexion angle (*d* = 0.389, 95% CI, 0.118–0.660, *p* = 0.006) and peak knee abduction angle (*d* = 0.339, 95% CI, 0.189–0.489, *p* < 0.001) during unanticipated sidestep cutting compared with anticipated cutting. For knee kinetics, unanticipated sidestep cutting resulted in greater peak knee abduction moment (*d* = 1.481, 95% CI, 0.857–2.104, *p* < 0.001). In contrast, no significant differences were observed for peak ATSF (*d* = 0.277, 95% CI, -0.051–0.604, *p* = 0.096), peak vGRF (*d* = -0.161, 95% CI, -0.527–0.205, *p* = 0.384), and vLR (*d* = 0.087, 95% CI, -0.036–0.209, *p* = 0.165).

**Table 2:** Comparison of approach velocity and biomechanical variables between anticipated and unanticipated sidestep cutting.

Variables	Value	Cohen’s *d* (95% CI)	*p-*value
Approach speed (m/s)			
Anticipated	4.48 ± 0.17	-0.251 (-0.687**–**0.185)	0.252
Unanticipated	4.44 ± 0.16		
Peak flexion angle (+) (°)			
Anticipated	44.55 ± 4.88	0.389 (0.118–0.660)	0.006
Unanticipated	46.63 ± 5.62		
Peak abduction angle (+) (°)			
Anticipated	-2.04 ± 8.79	0.339 (0.189–0.489)	< 0.001
Unanticipated	1.06 ± 9.34		
Peak abduction moment (+) (N·m·kg^-^¹)			
Anticipated	1.01 ± 0.27	1.481 (0.857–2.104)	< 0.001
Unanticipated	1.47 ± 0.34		
Peak ATSF (+) (N·kg^-^¹)			
Anticipated	6.00 ± 2.22	0.277 (-0.051–0.604)	0.096
Unanticipated	6.59 ± 2.04		
Peak vGRF (BW)			
Anticipated	2.27 ± 0.24	-0.161 (-0.527–0.205)	0.384
Unanticipated	2.21 ± 0.36		
vLR (BW·s^-^¹)			
Anticipated	51.52 ± 18.89	0.087 (-0.036–0.209)	0.165
Unanticipated	53.22 ± 19.92		

ATSF, anterior tibial shear force; vGRF, vertical ground reaction force; BW, body weight; vLR, vertical loading rate.

### 3.2 Moderating effects of cognitive performance on knee biomechanics

#### 3.2.1 Moderating effect of reaction time on knee biomechanics

**Figure 3:**
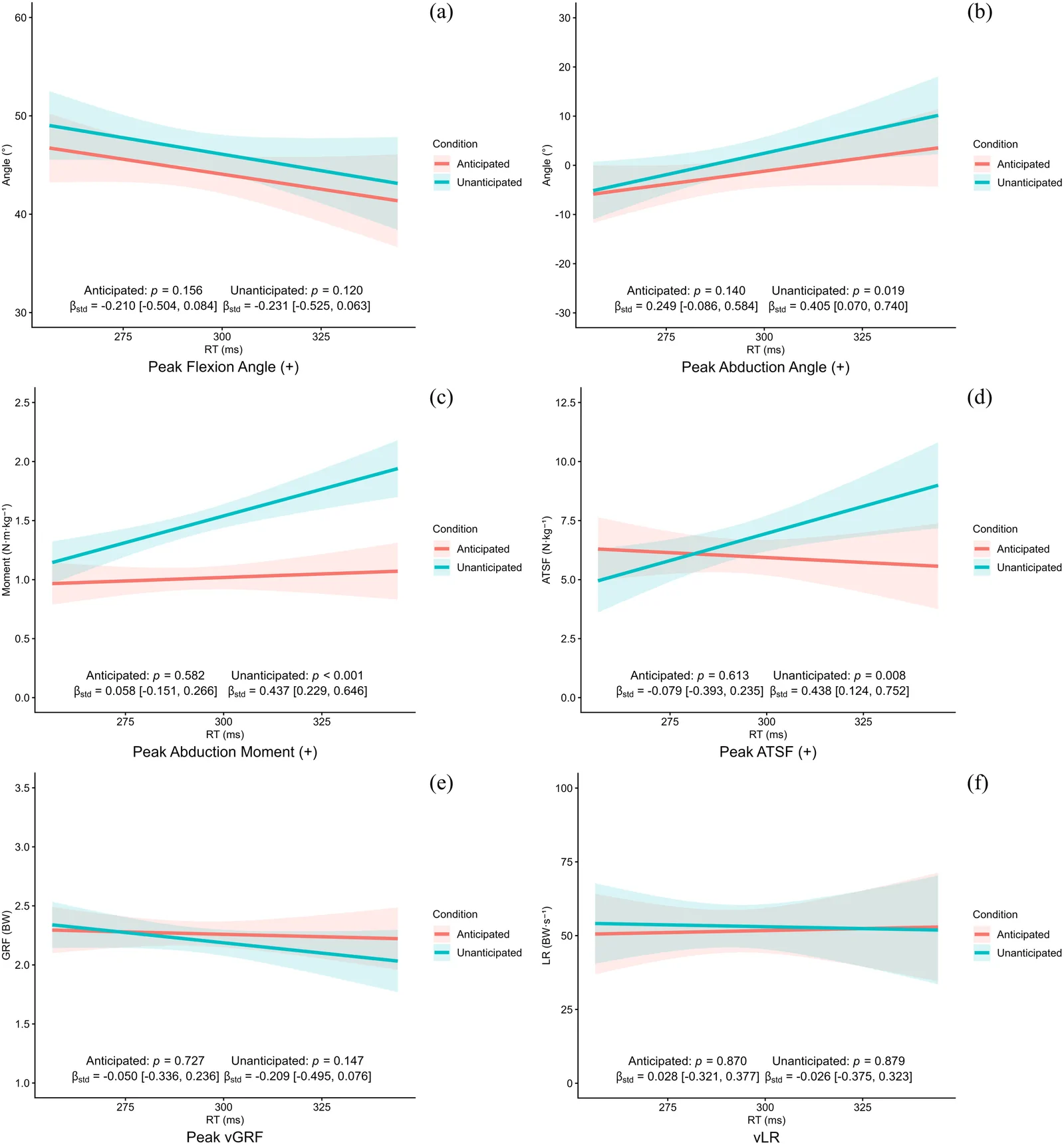
Simple slope analyses examining the effects of RT on knee biomechanics during anticipated and unanticipated sidestep cutting. **(a)** Peak flexion angle; **(b)** Peak abduction angle; **(c)** Peak abduction moment; **(d)** Peak ATSF; **(e)** Peak vGRF; **(f)** vLR. RT, reaction time; ATSF, anterior tibial shear force; vGRF, vertical ground reaction force; vLR, vertical loading rate.

Significant anticipation × RT interactions were observed for peak abduction angle (*β*_std_ = 0.156, 95% CI, 0.039–0.274, *p* = 0.031), peak abduction moment (*β*_std_ = 0.380, 95% CI, 0.168**–**0.592, *p* < 0.001), and peak ATSF (*β*_std_ = 0.517, 95% CI, 0.350–0.684, *p* < 0.001) (**Table 3**). In contrast, no significant anticipation × RT interactions were observed for peak flexion angle (*β*_std_ = -0.021, 95% CI, -0.228–0.187, *p* = 0.870), peak vGRF (*β*_std_ = -0.160, 95% CI, -0.389–0.070, *p* = 0.328), and vLR (*β*_std_ = -0.054, 95% CI, -0.206–0.097, *p* = 0.657). Simple slope analyses indicated that RT was positively associated with peak knee abduction angle (*β*_std_ = 0.405, 95% CI, 0.070–0.740, *p* = 0.019), peak knee abduction moment (*β*_std_ = 0.437, 95% CI, 0.229–0.646, *p* < 0.001), and peak ATSF (*β*_std_ = 0.438, 95% CI, 0.124–0.752, *p* = 0.008) during unanticipated sidestep cutting (**Figure 3**). Model diagnostic assessments indicated that all LMMs converged successfully, showed no evidence of singular fits, and yielded small maximum relative gradients. Residual skewness and excess kurtosis suggested no substantial departures from normality (**Supplementary Table 1**).

**Table 3:** LMMs examining RT on knee biomechanics during anticipated and unanticipated sidestep cutting.

Variables	Anticipation		RT		Anticipation × RT	
	*β*_std_ (95% CI)	*p-*value^a^	*β*_std_ (95% CI)	*p-*value^a^	*β*_std_ (95% CI)	*p-*value^a^
Peak flexion angle	0.321 (0.115**–**0.528)	0.013	-0.210 (-0.494**–**0.074)	0.328	-0.021 (-0.228**–**0.187)	0.870
Peak abduction angle	0.324 (0.207**–**0.441)	< 0.001	0.249 (-0.073**–**0.570)	0.328	0.156 (0.039**–**0.274)	0.031
Peak abduction moment	0.995 (0.784**–**1.207)	< 0.001	0.058 (-0.146**–**0.261)	0.582	0.380 (0.168**–**0.592)	< 0.001
Peak ATSF	0.252 (0.086**–**0.419)	0.013	-0.079 (-0.381**–**0.224)	0.766	0.517 (0.350**–**0.684)	< 0.001
Peak vGRF	-0.136 (-0.365**–**0.093)	0.408	-0.050 (-0.327**–**0.228)	0.839	-0.160 (-0.389**–**0.070)	0.328
vLR	0.080 (-0.071**–**0.231)	0.452	0.028 (-0.307**–**0.363)	0.870	-0.054 (-0.206**–**0.097)	0.657

^a^ *P*-values for peak knee abduction moment are unadjusted; all other *p*-values are adjusted using the Benjamini–Hochberg false discovery rate procedure.

RT, reaction time; *β*_std_, standardized regression coefficients; ATSF, anterior tibial shear force; vGRF, vertical ground reaction force; vLR, vertical loading rate.

#### 3.2.2 Moderating effect of commission error rate on knee biomechanics

**Figure 4:**
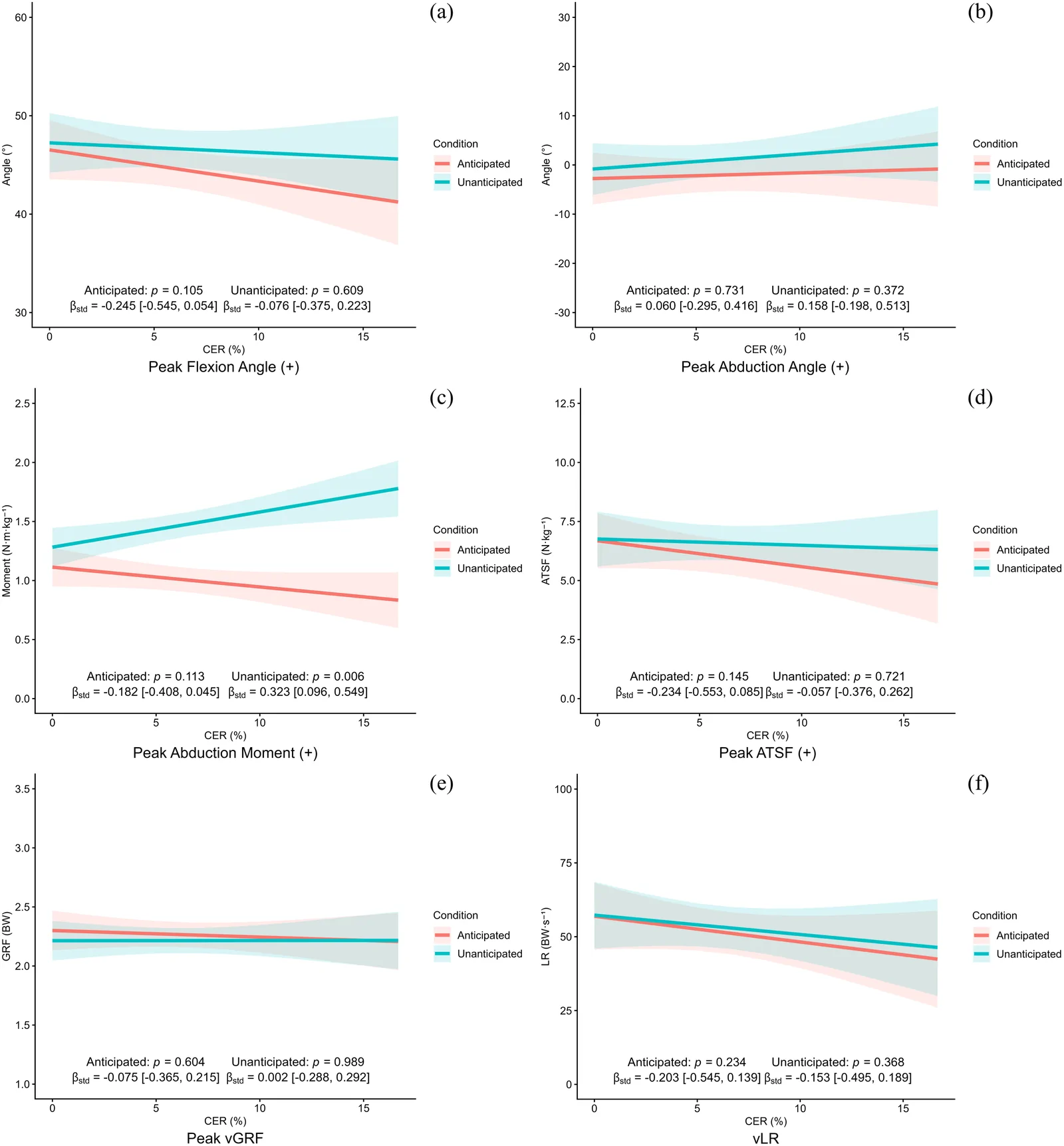
Simple slope analyses examining the effects of CER on knee biomechanics during anticipated and unanticipated sidestep cutting. **(a)** Peak flexion angle; **(b)** Peak abduction angle; **(c)** Peak abduction moment; **(d)** Peak ATSF; **(e)** Peak vGRF; **(f)** vLR. CER, commission error rate; ATSF, anterior tibial shear force; vGRF, vertical ground reaction force; vLR, vertical loading rate.

Significant anticipation × CER interactions were observed for peak abduction moment (*β*_std_ = 0.504, 95% CI, 0.299–0.710, *p* < 0.001) (**Table 4**). In contrast, no significant anticipation × CER interactions were observed for peak flexion angle (*β*_std_ = 0.169, 95% CI, -0.037–0.375, *p* = 0.238), peak abduction angle (*β*_std_ = 0.097, 95% CI, -0.022–0.217, *p* = 0.238), peak ATSF (*β*_std_ = 0.177, 95% CI, -0.007–0.362, *p* = 0.230), peak vGRF (*β*_std_ = 0.077, 95% CI, -0.154–0.308, *p* = 0.580), and vLR (*β*_std_ = 0.050, 95% CI, -0.101–0.201, *p* = 0.580). Simple slope analyses indicated that CER was positively associated with peak abduction moment (*β*_std_ = 0.323, 95% CI, 0.096–0.549, *p* = 0.006) during unanticipated sidestep cutting (**Figure 4**). Model diagnostic assessments indicated that all LMMs converged successfully, showed no evidence of singular fits, and yielded small maximum relative gradients. Residual skewness and excess kurtosis suggested no substantial departures from normality (**Supplementary Table 2**).

**Table 4:** LMMs examining CER on knee biomechanics during anticipated and unanticipated sidestep cutting.

Variables	Anticipation		CER		Anticipation × CER	
	*β*_std_ (95% CI)	*p-*value^a^	*β*_std_ (95% CI)	*p-*value^a^	*β*_std_ (95% CI)	*p-*value^a^
Peak flexion angle	0.321 (0.116**–**0.526)	0.023	–0.245 (–0.535**–**0.044)	0.238	0.169 (–0.037**–**0.375)	0.238
Peak abduction angle	0.324 (0.205–0.443)	< 0.001	0.060 (–0.281–0.402)	0.731	0.097 (–0.022–0.217)	0.238
Peak abduction moment	0.995 (0.790–1.200)	< 0.001	–0.182 (–0.402–0.038)	0.113	0.504 (0.299–0.710)	< 0.001
Peak ATSF	0.252 (0.068–0.436)	0.040	–0.234 (–0.542–0.073)	0.272	0.177 (–0.007–0.362)	0.230
Peak vGRF	–0.136 (–0.366–0.094)	0.371	–0.075 (–0.356–0.206)	0.647	0.077 (–0.154–0.308)	0.580
vLR	0.080 (–0.071–0.231)	0.410	–0.203 (–0.532–0.125)	0.371	0.050 (–0.101–0.201)	0.580

^a^ *P*-values for peak knee abduction moment are unadjusted; all other *p*-values are adjusted using the Benjamini–Hochberg false discovery rate procedure.

CER, commission error rate; *β*_std_, standardized regression coefficients; ATSF, anterior tibial shear force; vGRF, vertical ground reaction force; vLR, vertical loading rate.

#### 3.2.3 Moderating effect of omission error rate on knee biomechanics

**Figure 5:**
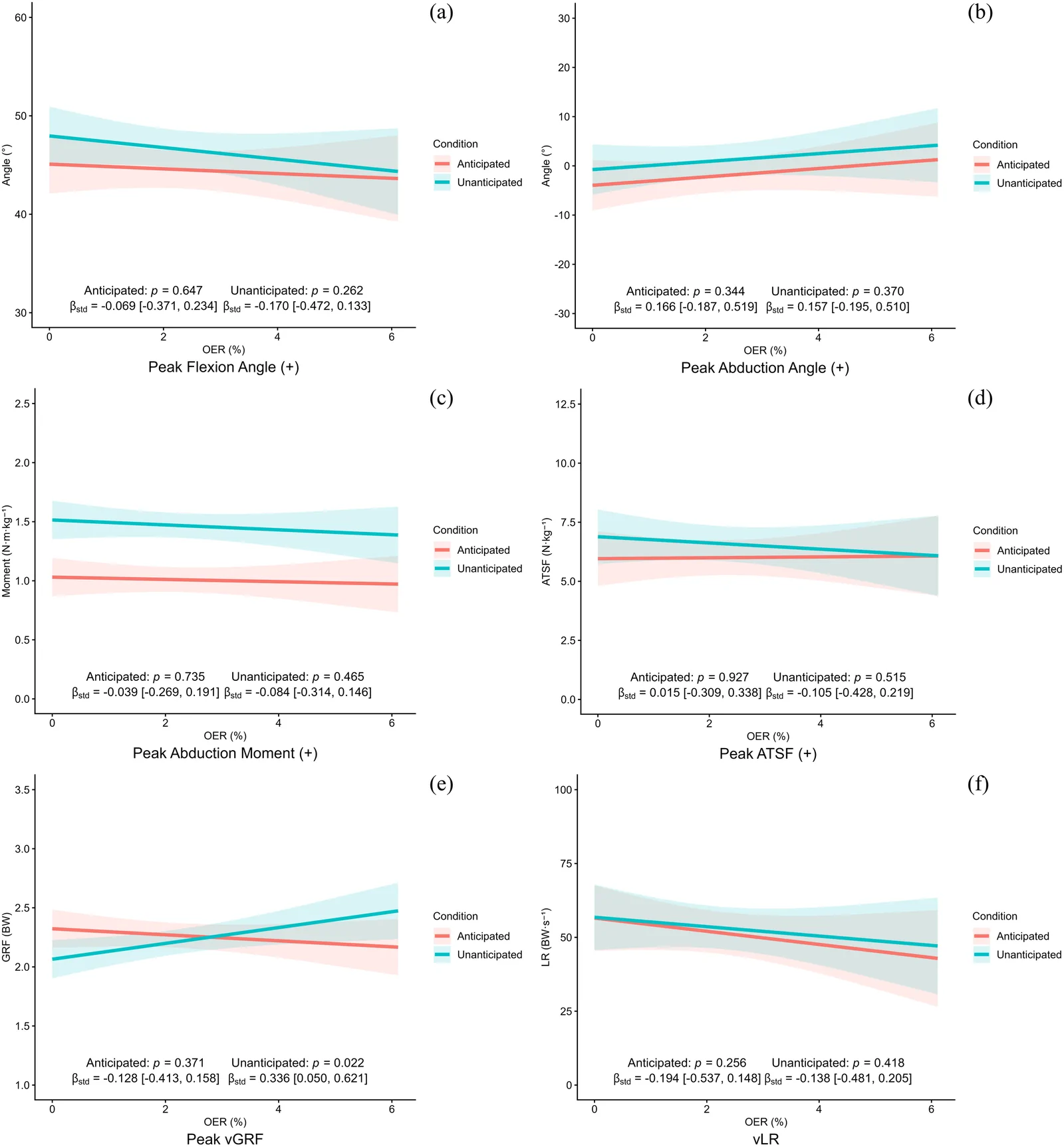
Simple slope analyses examining the effects of OER on knee biomechanics during anticipated and unanticipated sidestep cutting. **(a)** Peak flexion angle; **(b)** Peak abduction angle; **(c)** Peak abduction moment; **(d)** Peak ATSF; **(e)** Peak vGRF; **(f)** vLR. OER, omission error rate; ATSF, anterior tibial shear force; vGRF, vertical ground reaction force; vLR, vertical loading rate.

Significant anticipation × OER interactions were observed for peak vGRF (*β*_std_ = 0.463, 95% CI, 0.245–0.682, *p* < 0.001) (**Table 5**). In contrast, no significant anticipation × OER interactions were observed for peak flexion angle (*β*_std_ = -0.101, 95% CI, -0.308–0.106, *p* = 0.506), peak abduction angle (*β*_std_ = -0.008, 95% CI, -0.129–0.112, *p* = 0.927), peak abduction moment (*β*_std_ = -0.045, 95% CI, -0.266–0.176, *p* = 0.688), peak ATSF (*β*_std_ = -0.120, 95% CI, -0.305–0.066, *p* = 0.506), and vLR (*β*_std_ = 0.056, 95% CI, -0.095–0.207, *p* = 0.584). Simple slope analyses indicated that OER was positively associated with peak vGRF (*β*_std_ = 0.336, 95% CI, 0.050–0.621, *p* = 0.022) during unanticipated sidestep cutting (**Figure 5**). Model diagnostic assessments indicated that all LMMs converged successfully, showed no evidence of singular fits, and yielded small maximum relative gradients. Residual skewness and excess kurtosis suggested no substantial departures from normality (**Supplementary Table 3**).

**Table 5:** LMMs examining OER on knee biomechanics during anticipated and unanticipated sidestep cutting.

Variables	Anticipation		OER		Anticipation × OER	
	*β*_std_ (95% CI)	*p-*value^a^	*β*_std_ (95% CI)	*p-*value^a^	*β*_std_ (95% CI)	*p-*value^a^
Peak flexion angle	0.321 (0.115–0.528)	0.150	-0.069 (-0.361–0.223)	0.747	-0.101 (-0.308–0.106)	0.506
Peak abduction angle	0.324 (0.204–0.444)	< 0.001	0.166 (-0.172–0.504)	0.506	-0.008 (-0.129–0.112)	0.927
Peak abduction moment	0.995 (0.775–1.215)	< 0.001	-0.039 (-0.263–0.185)	0.735	-0.045 (-0.266–0.176)	0.688
Peak ATSF	0.252 (0.067–0.438)	0.031	0.015 (-0.297–0.327)	0.927	-0.120 (-0.305–0.066)	0.506
Peak vGRF	-0.136 (-0.354–0.082)	0.506	-0.128 (-0.404–0.149)	0.506	0.463 (0.245–0.682)	< 0.001
vLR	0.080 (-0.071–0.231)	0.506	-0.194 (-0.524–0.135)	0.506	0.056 (-0.095–0.207)	0.584

^a^ *P*-values for peak knee abduction moment are unadjusted; all other *p*-values are adjusted using the Benjamini–Hochberg false discovery rate procedure.

OER, omission error rate; *β*_std_, standardized regression coefficients; ATSF, anterior tibial shear force; vGRF, vertical ground reaction force; vLR, vertical loading rate.

## 4 Discussion

Overall, unanticipated cutting resulted in greater peak knee flexion angle, peak knee abduction angle, and peak knee abduction moment than anticipated cutting, reflecting distinct biomechanical responses across the sagittal and frontal planes. These responses were partially moderated by individual differences in cognitive performance. Specifically, slower RT was associated with greater peak knee abduction angle, peak knee abduction moment, and peak ATSF during unanticipated cutting. Higher CER was associated with greater peak knee abduction moment, whereas higher OER was associated with greater peak vGRF. These findings partially support our hypothesis and indicate that different aspects of cognitive performance may be associated with distinct biomechanical responses under unanticipated conditions.

Compared with pre-planned and controlled activities, musculoskeletal injuries are more likely to occur during open-skill sports involving competitive encounters and multitasking demands (Theisen et al., 2013; Ebner et al., 2025). In soccer, athletes are required to rapidly process external information (e.g., ball position, opponents, and teammates) and make appropriate decisions while simultaneously executing sport-specific movements (Mai et al., 2026). The increased cognitive demands associated with decision complexity, dual-task integration, stimulus responsiveness, uncertainty, and movement coordination in response to external targets may compromise neuromuscular control (Grooms and Onate, 2016). From a neurocognitive perspective, such cognitive–motor dual-task situations may lead to overlapping cortical activation and competition for limited attentional resources, thereby impairing the performance of one or both tasks (Kahneman, 1973; Rubin and Meiran, 2005). Indeed, ACL injuries frequently occur during rapid, externally directed movements under severe time constraints (Mai et al., 2026).

Consistent with this notion, the present findings showed that highly trained soccer players exhibited greater knee flexion angle, knee abduction angle, and knee abduction moment during unanticipated compared with anticipated sidestep cutting. In particular, greater knee abduction angle and abduction moment are recognized as key biomechanical predictors of ACL injury risk (Hewett et al., 2005). Kristianslund et al. (2014) reported that suboptimal cutting techniques, especially greater knee abduction at IC, increased knee abduction moment by approximately 19%. Furthermore, quantitative *in vitro* models have demonstrated that increased abduction loading can elevate peak ACL strain to approximately 1.5 times baseline levels (Bates et al., 2019). Interestingly, greater knee flexion angle is generally regarded as a protective strategy that may reduce ACL loading (Yu and Garrett, 2007). One possible explanation is that the severe time constraints during unanticipated sidestep cutting reduced the use of feedforward control mechanisms, thereby decreasing pre-activation of knee musculature and joint stiffness before IC (Taube et al., 2012). However, the peak knee flexion angle examined in the present study represented the maximum value during the WA phase rather than knee flexion at IC or at a specific early post-contact time point. Its potential protective interpretation should therefore be considered cautiously. Notably, this finding differs from previous studies focusing on single-leg landing tasks, which generally reported sagittal-plane impairments under increased cognitive load (Jiménez-Martínez et al., 2026; Abe et al., 2022; Dai et al., 2018). Beyond differences in movement type, single-leg landing studies often employ dual-task paradigms or distractors to elevate cognitive load, whereas sidestep cutting studies—including the present study—typically manipulate cognitive demands via directional uncertainty introduced by randomly presented visual cues (Jiménez-Martínez et al., 2025a). These differences in cognitive task design may elicit distinct biomechanical responses, highlighting the importance of considering task-specific cognitive demands when interpreting the interaction between cognition and lower-limb biomechanics.

Importantly, individual cognitive performance moderated the peak ATSF and peak vGRF responses, despite the absence of significant group-level differences in peak ATSF, peak vGRF, and vLR between the anticipated and unanticipated conditions. Specifically, slower RT was associated with greater peak ATSF, whereas higher OER was associated with greater peak vGRF during unanticipated cutting. These findings are partially supported by Shibata et al (Shibata et al., 2018), who reported that female athletes with poorer cognitive performance exhibited greater quadriceps activation relative to the hamstrings during unanticipated cutting maneuvers. Such a neuromuscular strategy may contribute to greater quadriceps-driven anterior tibial shear loading, a key biomechanical mechanism underlying increased ACL strain (Yu and Garrett, 2007). However, given the reported biomechanical and neuromuscular differences between male and female athletes during cutting tasks (Jonasson et al., 2023; Ghasemi et al., 2023), together with the fact that muscle activity was not directly assessed in the present study, this proposed mechanism should be interpreted cautiously. Nevertheless, the observed increases in ATSF and knee abduction moment under impulsive axial compression are consistent with the well-established multiplanar mechanism of ACL injury, which produces greater peak ACL strain than loading in a single plane (Kiapour et al., 2016; Koga et al., 2017). These findings suggest that poorer cognitive performance may be associated with greater ACL injury–related knee loading during unanticipated sidestep cutting.

Previous studies have explored multiple cognitive domains that may influence lower-extremity biomechanics, including attention, processing speed, working memory, and inhibitory control (Jiménez-Martínez et al., 2026; Bertozzi et al., 2023a, 2023; Giesche et al., 2020; Shibata et al., 2018; Jiménez-Martínez et al., 2025b). Increasing attention has been directed toward the role of inhibitory control (Jiménez-Martínez et al., 2025b, 2026; Gokeler et al., 2024; Giesche et al., 2020). In sport, inhibitory control refers to the ability to regulate or suppress automatic responses and impulses, thereby preventing the execution of inappropriate actions (Friedman and Miyake, 2004). For instance, when an opponent unexpectedly changes direction, a player must inhibit the initially planned movement and rapidly execute an alternative cutting maneuver based on the updated environmental information. Poor inhibitory control may impair the ability to effectively attenuate impact loads during dynamic movements, leading to altered movement mechanics and increased ACL injury risk (Gokeler et al., 2020). It was reported that neurocognitive errors related to motor response inhibition and attentional inhibition are commonly observed during non-contact ACL injury situations in professional male soccer players (Gokeler et al., 2024). Thus, the observed associations may reflect the contribution of baseline inhibitory control within a broader cognitive–motor process required for unanticipated cutting.

RT has consistently been identified as one of the most important cognitive predictors of musculoskeletal injury risk (Bertozzi et al., 2023a; Jiménez-Martínez et al., 2026; McDonald et al., 2019). Greater peak knee abduction angle and moment have been associated with baseline RT (Bertozzi et al., 2023a). Similarly, a recent study (Jiménez-Martínez et al., 2026) further showed that individuals with poorer RT exhibited more unfavorable biomechanical patterns as cognitive demands increased during single-leg landing. Consistent with these findings, RT emerged as the strongest moderator of ACL injury–related biomechanics in the present study, with the model including RT and its interaction with anticipation accounting for approximately 34% of the variance in peak knee abduction moment (**Supplementary Table 1**). Under unanticipated conditions, poorer RT may limit athletes’ ability to rapidly process unexpected external information, thereby restricting the appropriate activation of feedforward control mechanisms and delaying neuromuscular responses (Morris and Christie, 2020; Mornieux et al., 2014). This may increase reliance on passive joint structures to absorb impact loads, ultimately increasing ACL injury risk (Zhang et al., 2018). This view is further supported by a previous study (Mornieux et al., 2014), which demonstrated that as ART decreased during unanticipated sidestep cutting, the knee abduction moment and vGRF increased. Moreover, the ACL injury window in soccer typically occurs within 40–80 ms after IC (Zago et al., 2024), whereas feedback-driven responses generally require 150–200 ms to respond to risky landing mechanics (Koga et al., 2017). This temporal mismatch further highlights the important role of RT in ACL injury.

### 4.1 Implications for future research

The present findings may have implications for future ACL injury mitigation research in soccer. First, the observed biomechanical differences between anticipated and unanticipated sidestep cutting suggest that future training studies should examine whether incorporating uncertainty and decision-making demands better reflects the movement challenges encountered during match play. Second, cognitive performance may warrant further investigation as a potential component of multifactorial ACL injury screening; however, prospective studies are required to determine whether cognitive measures improve the prediction of actual injury occurrence. Third, intervention studies should evaluate whether perceptual-cognitive or “brain endurance” training, in addition to traditional neuromuscular training, can improve movement adaptability and ACL injury–related biomechanics during cognitively demanding sporting actions.

### 4.2 Limitations

Several limitations of the present study should be acknowledged. First, the unanticipated cutting task was induced using visual stimuli presented under laboratory conditions, which may not fully replicate the perceptual and decision-making complexity encountered during actual match play. Second, because the study included only highly trained (Tier 3), right-leg-dominant male soccer players, the generalizability of the findings to athletes at other competitive levels, female athletes, and individuals with different limb-dominance profiles remains uncertain. Third, baseline cognitive performance was assessed at a single time point. Given that cognitive function may fluctuate in response to factors such as training load, competitive schedules, and recovery status, future research should investigate whether longitudinal changes in cognition are associated with alterations in movement biomechanics. Fourth, the *a priori* sample-size calculation was not specifically designed to detect anticipation × cognitive-metric interaction effects; therefore, the moderation findings should be interpreted as exploratory and require confirmation in larger studies. Fifth, only successful cutting trials were included in the biomechanical analysis. This approach may have excluded relevant cognitive–motor performance reflected in directional errors, loss of balance, or failure to meet the predefined trial criteria, thereby introducing a potential selection effect. Finally, the present study focused solely on inhibitory control as assessed by the Go/No-Go task. However, the unanticipated sidestep cutting task likely involved additional cognitive processes, including choice reaction, visuospatial processing, stimulus–response mapping, cognitive flexibility, and working memory. These domains were not directly assessed and warrant further investigation.

## 5 Conclusion

In conclusion, unanticipated sidestep cutting altered multiple ACL injury–related biomechanical variables across the sagittal and frontal planes in highly trained male soccer players. More importantly, baseline cognitive performance, particularly RT, partially moderated several ACL injury–related biomechanical responses under unanticipated conditions. These findings provide new insights into the association between baseline cognitive performance and ACL injury–related biomechanical responses during cognitive-motor tasks. However, larger replication studies and prospective investigations are required before these measures can be considered for injury-risk prediction or athlete screening.

## Data Availability

The raw data supporting the conclusions of this article will be made available by the authors, without undue reservation.
