# Supplementary material for: Baseline cognitive performance moderates ACL injury–related knee biomechanics during unanticipated sidestep cutting in highly trained male soccer players
Source: Front Physiol. 2026 Aug 18;17:1911093. doi: 10.3389/fphys.2026.1911093 (PMC13529485; doi:10.3389/fphys.2026.1911093)
Supplement: Supplementary file 1 [file Table1.docx]

Supplementary Table S1. Model fit and diagnostic statistics for LMMs examining RT

| Valuables | *R*^2^_m_ | *R*^2^_c_ | Convergence code | Singular fit | Max relative gradient | Residual skewness | Residual excess kurtosis |
| --- | --- | --- | --- | --- | --- | --- | --- |
| Peak flexion angle | 0.072 | 0.516 | 0 | No | < 0.001 | -0.404 | 1.525 |
| Peak abduction angle | 0.133 | 0.846 | 0 | No | < 0.001 | 0.155 | 0.788 |
| Peak abduction moment | 0.340 | 0.484 | 0 | No | < 0.001 | 0.349 | 0.023 |
| Peak ATSF | 0.110 | 0.689 | 0 | No | < 0.001 | -0.429 | 1.515 |
| Peak vGRF | 0.027 | 0.406 | 0 | No | < 0.001 | 0.131 | -0.323 |
| vLR | 0.002 | 0.747 | 0 | No | < 0.001 | -0.173 | 1.410 |

Abbreviations: RT, reaction time; *β*_std_, standardized regression coefficients; *R*^2^_m_, marginal *R*²; *R*^2^_c_, conditional *R*²; ATSF, anterior tibial shear force; vGRF, vertical ground reaction force; vLR, vertical loading rate.

Supplementary Table S2. Model fit and diagnostic statistics for LMMs examining CER

| Valuables | *R*^2^_m_ | *R*^2^_c_ | Convergence code | Singular fit | Max relative gradient | Residual skewness | Residual excess kurtosis |
| --- | --- | --- | --- | --- | --- | --- | --- |
| Peak flexion angle | 0.057 | 0.525 | 0 | No | < 0.001 | -0.416 | 1.303 |
| Peak abduction angle | 0.039 | 0.843 | 0 | No | < 0.001 | 0.127 | 0.978 |
| Peak abduction moment | 0.311 | 0.519 | 0 | No | < 0.001 | 0.406 | 0.721 |
| Peak ATSF | 0.043 | 0.620 | 0 | No | < 0.001 | -0.246 | 0.698 |
| Peak vGRF | 0.007 | 0.401 | 0 | No | < 0.001 | 0.094 | -0.493 |
| vLR | 0.032 | 0.746 | 0 | No | < 0.001 | -0.203 | 1.621 |

Abbreviations: CER, commission error rate; *β*_std_, standardized regression coefficients; *R*^2^_m_, marginal *R*²; *R*^2^_c_, conditional *R*²; ATSF, anterior tibial shear force; vGRF, vertical ground reaction force; vLR, vertical loading rate.

Supplementary Table S3. Model fit and diagnostic statistics for LMMs examining OER

| Valuables | *R*^2^_m_ | *R*^2^_c_ | Convergence code | Singular fit | Max relative gradient | Residual skewness | Residual excess kurtosis |
| --- | --- | --- | --- | --- | --- | --- | --- |
| Peak flexion angle | 0.041 | 0.520 | 0 | No | < 0.001 | -0.351 | 1.374 |
| Peak abduction angle | 0.050 | 0.840 | 0 | No | < 0.001 | 0.089 | 0.736 |
| Peak abduction moment | 0.248 | 0.444 | 0 | No | < 0.001 | 0.369 | 0.05 |
| Peak ATSF | 0.021 | 0.616 | 0 | No | < 0.001 | -0.291 | 0.732 |
| Peak vGRF | 0.067 | 0.462 | 0 | No | < 0.001 | 0.083 | -0.442 |
| vLR | 0.041 | 0.520 | 0 | No | < 0.001 | -0.351 | 1.374 |

Abbreviations: OER, omission error rate; *β*_std_, standardized regression coefficients; *R*^2^_m_, marginal *R*²; *R*^2^_c_, conditional *R*²; ATSF, anterior tibial shear force; vGRF, vertical ground reaction force; vLR, vertical loading rate.
